# Material Selection Process for Acoustic and Vibration Applications Using the Example of a Plate Resonator

**DOI:** 10.3390/ma15082935

**Published:** 2022-04-18

**Authors:** Moritz Neubauer, Felix Schwaericke, Vincent Radmann, Ennes Sarradj, Niels Modler, Martin Dannemann

**Affiliations:** 1 Institute of Lightweight Engineering and Polymer Technology (ILK), Technische Universität Dresden, Holbeinstraße 3, 01307 Dresden, Germany; niels.molder@tu-dresden.de; 2 Institute of Fluid Dynamics and Engineering Acoustics, Technische Universität Berlin, Einsteinufer 25, 10587 Berlin, Germany; schwaericke@tu-berlin.de (F.S.); vincent.radmann@tu-berlin.de (V.R.); ennes.sarradj@tu-berlin.de (E.S.); 3 Faculty of Automotive Engineering, Institute of Energy and Transport Engineering, Westsächsische Hochschule Zwickau, Scheffelstraße 39, 08012 Zwickau, Germany; martin.dannemann@fh-zwickau.de

**Keywords:** material selection method, plate resonator, thermoplastic, model, acoustic liner

## Abstract

In this work, a new method for selecting suitable materials is presented. This method has a high potential for a variety of engineering applications, such as the design of sound-absorbing and vibration-loaded structures, where a large number of different requirements have to be met. The method is based on the derivation of functional dependencies of selected material parameters. These dependencies can be used in parameter studies to consider parameter combinations that lie in the range of real existing and targeted material groups. This allows the parameter space to be reduced, the calculation to be accelerated, and suitable materials to be (pre-)selected for the respective application, which contributes to a more target-oriented design. The method is applied to the example of a plate resonator. For this purpose, a semi-analytical model is implemented to calculate the transmission loss as well as the reflected and dissipated sound power of plate silencers, taking into account the influence of flow velocity and fluid temperature on the performance of plate silencers.

## 1. Introduction

Numerous methodologies have been introduced that aim to provide a guiding structure in the process of selecting the optimal material for a particular application [1]. The frameworks described by Ashby et al. [2] and Van Kesteren et al. [3] coincide in the concept that material selection includes the basic steps of defining requirements and certain criteria, the screening of materials to generate a set of potential candidates, the comparison and subsequent ranking of suitable candidates, and the final step of selecting the optimal material. In this context, Ashby charts are often used for screening because they allow facile and convenient comparison of property ratios of groups of materials. The weighted property methods (WPM) as well as the multi-criteria decision (MCDM) are suitable methods to narrow down the number of potential materials [4,5,6,7,8]. In this context, MCDM techniques apply material selection decision matrices and criteria sensitivity analysis to enable a logical ranking of considered materials [9,10,11]. An alternative approach of supporting the decision process in material selection lies in the application of artificial intelligence [12,13,14]. The so-called expert systems consist of modules for knowledge acquisition, inference, as well as a user interface module that enables the simulation of human experts’ reasoning and decision making [15,16,17]. For the present work, the material property charts introduced by Ashby and Cebon [18] are used to identify functional dependencies of the relevant material properties in order to reduce the parameter space and obtain a coherent representation of the relationships that influence the target variable.

For materials used in complex applications, analytical models are often derived and subsequently used to relate the relevant parameters. However, depending on the complexity of the application, the target parameters may depend on a large number of material parameters whose actual influence on the target variable is unknown. This makes it rather difficult to identify the material parameters with significant influence on the target variable as well as suitable value ranges that can be used as a basis for material selection. In addition, some material parameters show dependencies that cannot be resolved. A typical example is the observation that a material generally cannot have both high stiffness and high material damping. This is a typical conflict of targets for many applications in the field of vibration and acoustics. Due to the vast number of relevant parameters, the modeling of a plate resonator (PR) is rather complex, leading to computational and time-intensive parameter studies in the context of an optimization. Therefore, a method is required to specify the stepwise model-based material selection for acoustic PR liners. Consequently, the narrowing of the parameter space and the identification of parameters with significant influence on the target variable through material group analysis enables a time- and resource-efficient material selection. In this context, the presented determination of a functional dependence between the material parameters allows a more decisive and coherent analysis of their effects on the target variable. The representation of the parameters of interest within a spectrum provides additional information that can support the choice of the formulated optimization goal and is a possible complement to the method of applying performance indices.

Plate silencers, that consist of expansion chambers fully covered with a plate, are especially used for attenuating low frequencies [19,20]. Although plate resonators are already used in practice, it turned out that the obtained experimental results and the practical experience with plate resonators agree with the established computational models only under restrictions [21]. The design of this type of resonance absorber is therefore considered complex and requires additional supporting measurements [22].

The most advanced model currently used for describing plate resonators is the one developed by Huang and Wang [23,24]. It is a semi-analytical model that describes the interaction of the plate with the underlying cavity and the duct above. Excited by a plane wave, the system of plate and cavity resonates, and the plate radiates sound back into the duct. This causes the radiated sound to overlap with the incoming sound, resulting in partial cancellation. In addition, part of the sound energy in this process is dissipated by internal losses in the material of the plate.

Plate silencers have many advantages to silencers made of porous material or perforated surfaces. They can be exposed to contaminated air, heat, frost, and humidity without being damaged. Due to their smooth surfaces, their flow resistance is comparatively low [25]. There are numerous applications where the advantageous properties of the smooth and robust sheet surface can be effectively utilized, e.g., as suspended transparent ceiling installations made of plastic films to meet high air quality requirements in clean rooms [22,26]. Furthermore, plate resonators are widely used as low-frequency tuned silencers in industrial ventilation systems as well as in the pipework of central air conditioning systems to reduce noise emissions [22,27]. In the automotive industry, they are used as engine compartment linings and in mufflers to attenuate the broadband and low-frequency sound emitted by the engine, while saving costs and installation space [24,28]. In this context, the performance of the plate silencer is significantly influenced by the material properties of the plate. Hence, it is important to find a suitable material for the specific application.

The aim of the presented work is to introduce a novel model-assisted methodology for material selection using the example of the plate silencer. After describing the general steps of the method for model-based material selection, the underlying model of a plate resonator as well as the relevant properties are introduced. Subsequently, the task of material selection is described considering functional dependencies among properties and the interaction between the selection process and the model. The functional dependency allows two material parameters to be reduced to one and subsequently visualize its effect on the target variable in a frequency spectrum. This enables the coherent analysis of the effect of the relevant material parameters on a target variable over a wide value range and the identification of practice-relevant trends. After deriving and presenting the general and applied method of model-based material selection, the derived results are discussed.

## 2. Materials and Methods

**Figure 1:**
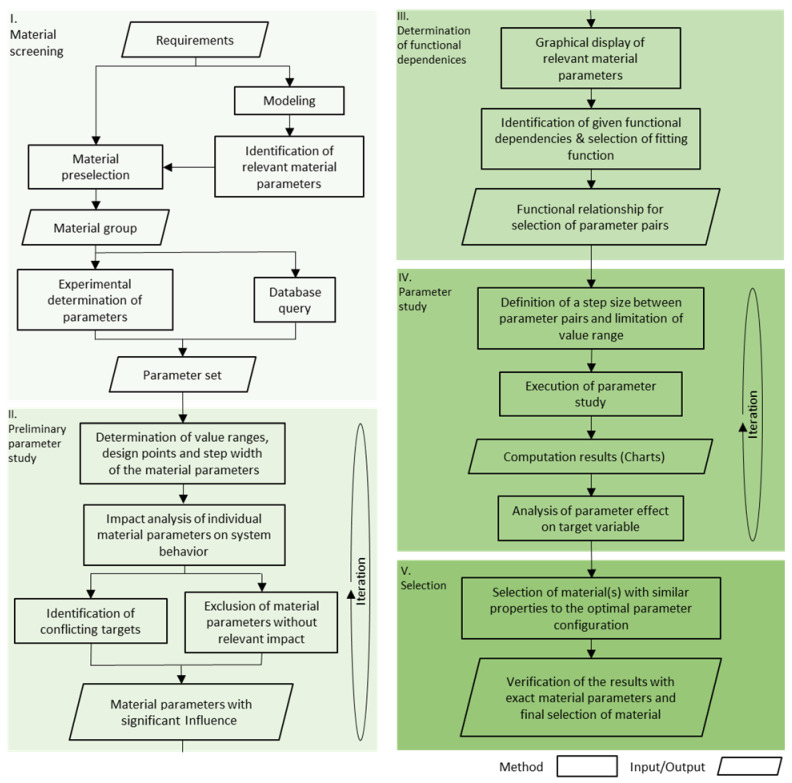
Methodology of model-based material selection for complex applications.

The generalized approach to model-based material selection for complex applications is described below. The methodology shown in Figure 1 is subsequently applied to the selection of the plate material for a plate resonator.

### 2.1. Material Screening

The starting point is the definition of requirements resulting from the intended application and the subsequent development of the model to describe the relationships and phenomena of the application. In the course of the modeling process, the relevant material parameters are identified. Together with the defined requirements, they enable pre-selection by excluding groups of materials due to certain specifications exceeding the limits of the associated properties [29,30]. In addition to model-specific parameters, this can include requirements arising from the product lifecycle, such as restrictions in the fields of manufacturing technologies or recycling. After the selection of suitable material groups, the fundamental parameter set(s) is/are established either by experimental characterization or database query.

### 2.2. Preliminary Parameter Study (Independent Variation)

The objective of the preliminary parameter study is a first exploration of the influence of particular material parameters. This enables the identification of parameters with a significant effect on the target variable in order to select them for a subsequent step of determining the functional dependencies. Here, a list (e.g., see Table 1) of parameters required to model the problem can be used. In order to narrow down the parameter space, a design point as well as a maximum range are defined. This is carried out by examining the distribution of property values of the targeted material group. For properties that scatter over a large range, a logarithmic step size is beneficial for analyzing its effect on the target variable. Starting from the design point, the parameter is then varied towards the defined limits. In case the material parameter and the target variable have a direct proportional relationship, this parameter is not considered further due to its comprehensible effect on the target variable. However, if the material parameter has a non-facile but contrary effect that leads to conflicting targets, this parameter has a relevant influence on the target variable that needs to be investigated in more detail.

### 2.3. Determination of Functional Dependence

The independent variation of properties in parameter studies leads to large areas in the parameter space not being represented by real materials. This unnecessary increase in parameter space increases computational resources time, and complicates the selection of appropriate materials. In order to perform the parameter studies in the range of existing materials, the identification of functional dependencies between the previously defined relevant material parameters using material parameter charts is a useful tool. For the representation and assessment, the relevant material parameter can therefore be plotted on a respective axis in charts [18]. The visualization can support the process of identification of functional dependencies of the selected material properties [29]. Depending on the size of the selected group of materials and the range of the properties in terms of magnitude, this can be performed in a linear single or double logarithmic representation. Subsequently, the resulting plots are checked for existing functional dependencies. After a relationship has been identified, a corresponding basis function is selected. Then, using suitable mathematical regression methods, the function that best describes the given dependencies is determined.

### 2.4. Parameter Study

Based on the functional relationship, derived in Section 2.3, value pairs can now be calculated to serve as input variables for the model-based parameter study. The step size between the pairs should be based on the step size determined in Section 2.3, to allow an analysis of the effect on the target variable. If unfavorable step sizes are chosen, it may be difficult to assess the effect of the parameter variation of the value pairs on the target variable. In this case, it is advisable to reduce the step size to make the effects on the target variable clear. Based on the observed trends on the target variable, a material could be selected. However, it may be required to apply a finer resolution over the entire parameter range in case more sections are of particular interest. In this way, the effects of the relevant parameters can be mapped across the entire value range. This assumes that the required computational effort remains feasible.

### 2.5. Selection

In the last step, a parameter combination is selected, that provides the desired result with respect to the target variable. Depending on the accuracy of the functional dependency, the material can be selected directly according to the identified parameter combination. In case the determined parameter configuration differs slightly from existing materials, the parameter combination of one or more materials that are in the target range can be analyzed and the desired material(s) can be selected. 

## 3. Application of the Presented Methodology for the Selection of a Plate Material for a Plate Resonator

In this section, a brief overview of the plate resonator model is provided before applying the presented material selection method to determine the plate material of the silencer.

### 3.1. Application—Plate Silencer

The methodology introduced in the present work is validated during the design process of an acoustic plate silencer, more specifically through the selection of its plate material. The requirements for the plate derive from the intended applications as a silencer as well as from the general environmental aspects of the product lifecycle.

**Figure 2:**
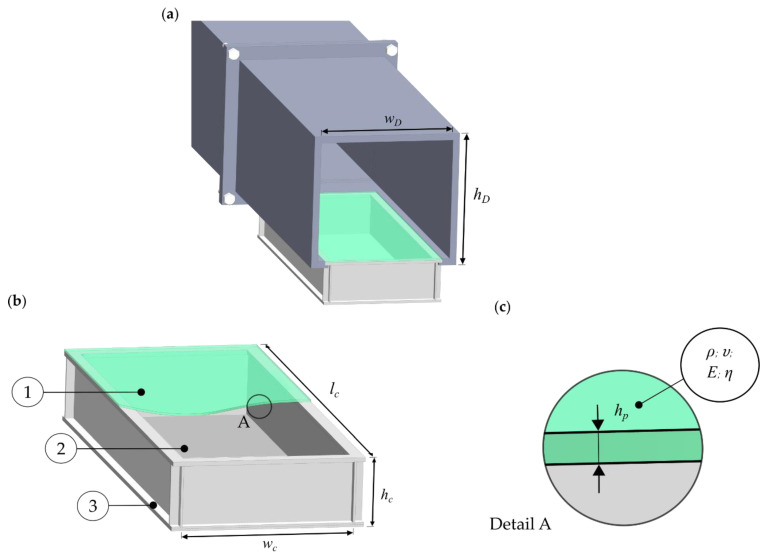
(**a**) Duct with defined height $h_{D}$, width $w_{D}$ and integrated resonator liner, (**b**) design of a plate resonators with ① its film layer ② the enclosed cavity, comprising the cell length $l_{C}$, the cell height $h_{c}$, the cell width $w_{c}$ and ③ the bottom layer. (**c**) Detailed view of the film with the relevant material properties, being density ρ, Young’s modulus E, Poisson ratio υ and the loss factor η as well as the film thickness $h_{p}$.

In general, the plate silencer can be used in ducts such as exhaust systems. A simple model of a plate silencer is shown in Figure 2. Here, a plate capable of oscillating (Figure 2, green) replaces the upper wall facing the duct. Behind this plate is a closed volume, called a cavity. As sound propagates along the duct, the plate is excited by the pressure difference above and below it. As a result, one part of the sound is reflected, and another part is dissipated by the vibration of the plate and its interaction with the cavity. To describe this process, a model developed by Huang and Wang is applied, describing a two-dimensional plate silencer with a simply supported plate [23,24]. In this context, the validity of this model was verified through the comparison of experimental results, that showed a satisfying level of accordance [31,32]. In addition, the validated model has been used in further parameter studies in the context of acoustic investigations, including the analysis of sandwich and composite plates [19,25,33]. Therefore, this model was chosen to demonstrate the material selection process of a plate silencer. To evaluate the performance of the plate silencer, the transmission loss can be used, which was set as the target variable for the present material selection process.

Therefore, the bending differential equation of the plate must be solved for its velocity. In this context, the right side of Equation (1) corresponds to the excitation of the plate. The sound fields above and below the plate can be represented by a kind of impedance for the duct $\underline{\underline{Z_{D}}}$, and the cavity $\underline{\underline{Z_{C}}}$ (see Figure 2). Additionally, such an impedance matrix can also be formulated for the plate ($\underline{\underline{L}}$), which contains the properties of the plate material. In order to solve the differential equation, a Galerkin method was used. Thus, with the impedance matrices of the duct cavity plate, and the incident sound $\underline{I}$, the following system of equations can be solved for the modal amplitude of the plate velocity, $\underline{v}$.

(1) $$\left(\underline{\underline{L}}+\underline{\underline{Z_{C}}}+\underline{\underline{Z_{D}}}\right)\underline{v}=-\underline{I}$$

To solve this equation, a modular implementation of the plate silencer model was coded in python. With the determined velocity, the radiated sound power of the plate can be calculated, allowing the transmitted and reflected sound power to be calculated as well. The transmission loss, representing the target variable, can be computed from the transmitted sound power $P_{\mathrm{trans}}$, and the incident sound power $P_{in}$, as follows:

(2) $$TL=-10\log\left|\frac{P_{trans}}{P_{in}}\right|.$$

The sound power dissipated due to the intrinsic damping behavior of the plate is derived from the difference between the incident sound power on one side and the reflected and transmitted sound power on the other side.

The python implementation of the plate silencer model allows to calculate single-sided and double-sided configurations. In the procedure described here, the single-sided configuration was applied (see Figure 2). The foundation of the computation of the transmission loss is the input parameters contained in the impedance matrices. As shown in Table 1, they are divided in geometry and material parameters.

Since the calculation was performed in two dimensions, the displayed width of the duct $w_{D}$, and the cavity $w_{C}$, is considered infinite. The dimensions of the resonator were chosen to create a silencer small enough to be used in a machine or vehicle. The purpose here was to demonstrate that a plate resonator can be used as a low-frequency silencer requiring very little space. At the same time, the small dimensions limit the number of modes to be damped in the system, which reduces the computation time and allows the simulation of more material configurations.

### 3.2. Material Selection Process

In the following section, the above-presented method is applied to the material selection of the plate of a plate silencer.

#### 3.2.1. Material Screening

The identification of a suitable material group is primarily based on the field of application. Depending on this, the goal in noise control is often to effectively attenuate low frequencies, since they are more difficult to suppress than high frequencies. Secondarily, the material identification process is influenced by the requirements of the design and manufacturing process. For the presented example of a plate silencer, the material group of thermoplastics and thermoplastic elastomers was chosen. Compared to, e.g., metals, these materials have a higher internal damping characteristic, which can lead to a higher overall damping of the silencer. In addition, they are easy to process, can be joined using a variety of joining techniques, and are relatively easy to manufacture, even in the desired low plate thicknesses.

#### 3.2.2. Preliminary Parameter Study

After the process of selecting the material group is completed, the second step is to conduct a preliminary parameter study for first exploration in order to assess the effect of the individual material parameters on the target variable. Therefore, the effect of the Young’s modulus, loss factor, and density on the transmission loss was analyzed. For this purpose, the value range, step size, and the design point were defined first. As described in Section 2.2 of the presented method, a logarithmic and linear step size was determined based on the value distribution of the material parameters for the selected material group. The parameter ranges and design points that were finally applied are listed in Table 1.

**Figure 3:**
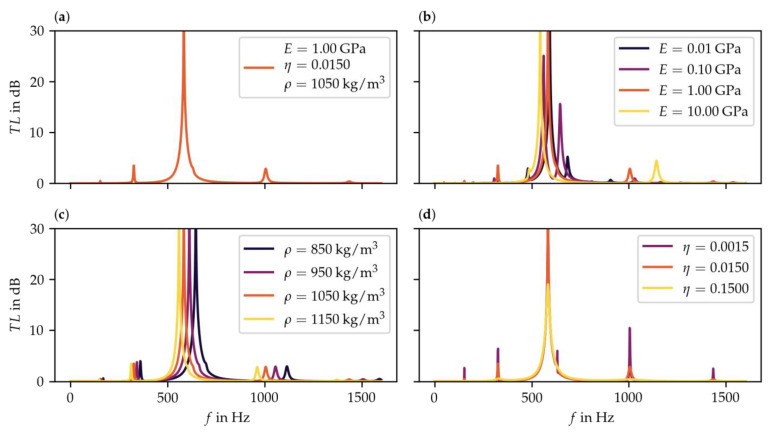
Results of the preliminary parameter study, (**a**) reference result of the design point, (**b**) variation of Young’s modulus E, (**c**) variation of density ρ, (**d**) variation of loss factor η.

Starting from the design point of the preliminary parameter study, the simulation leads to a reference spectrum of the transmission loss (see Figure 3a). From there, all three parameters were varied independently towards the defined limits. For the density and the loss factor, the effects were distinct and predictable. The density results in a relative change in frequency, with a higher density shifting the spectrum to lower frequencies (see Figure 3c). Since the value range of the density within the targeted material group is comparatively small and the mass of the plate can also be changed by varying the plate thickness, the influence of this parameter does not need to be investigated further, leaving the Young’s modulus and the loss factor for further investigations.

The loss factor broadens and reduces the transmission loss peaks in the spectrum. The effect is of different intensity for the main peak compared to the smaller peaks above and below it (see Figure 3d). Compared to the density, there exists a significantly larger value range for the loss factor within the material group of thermoplastics and thermoplastic elastomers. Therefore, and due to the fact that the loss factor has a higher qualitative as well as a more diverse effect on the transmission loss, further analysis of the parameter is required.

The influence of the Young’s modulus is as yet undetermined, since no clear effect can be observed. Based on the goal of low-frequency attenuation and analyzing the results shown in Figure 3b, it appears that high values for the Young’s modulus are preferable. Nevertheless, it seems that a more detailed study on the influence of the Young’s modulus, considering smaller step sizes, is necessary.

#### 3.2.3. Determination of Functional Dependencies

**Figure 4:**
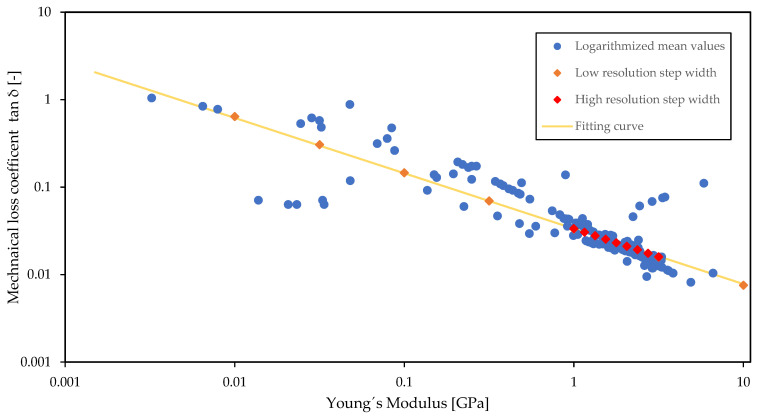
Mechanical loss factor over Young’s modulus of thermoplastic and thermoplastic elastomer polymers: the fitting curve represents the functional dependence between the two material parameters as well as the two sets of parameter pairs with different step sizes for the subsequent effect analysis (material data from [35]).

In order to reduce and maintain the parameter space in the range of existing thermoplastic polymers, the identification of a functional dependence between the relevant material parameters is targeted, as described in Section 2.3 of the introduced method. Since the preliminary parameter study implied the relevance of the Young´s modulus and the mechanical loss factor with regard to the target variable, these two material parameters were considered for the determination of a functional dependency. For the purpose of identifying a dependency, the corresponding pairs of values of the material parameters were analyzed in the form of a material property chart introduced by Ashby [34], varying the scales of both axes. In Figure 4, the logarithmic mean values of Young´s modulus and mechanical loss factor considering the material-envelopes of 211 representatives sub-classes of the material class of thermoplastics and thermoplastic elastomers were analyzed on a logarithmic scale for both axes [35]. The graphical display of the parameters reveals the linear dependency in a double logarithmic representation, leading to a polynomic interrelationship in linear space (see Figure 4).

Applying a polynomial basis function, the numerical relationship was established by linear regression in the form of the following equation:

(3) $$\eta =0.0335\cdot E^{-0.633}.$$

#### 3.2.4. Parameter Study

**Figure 5:**
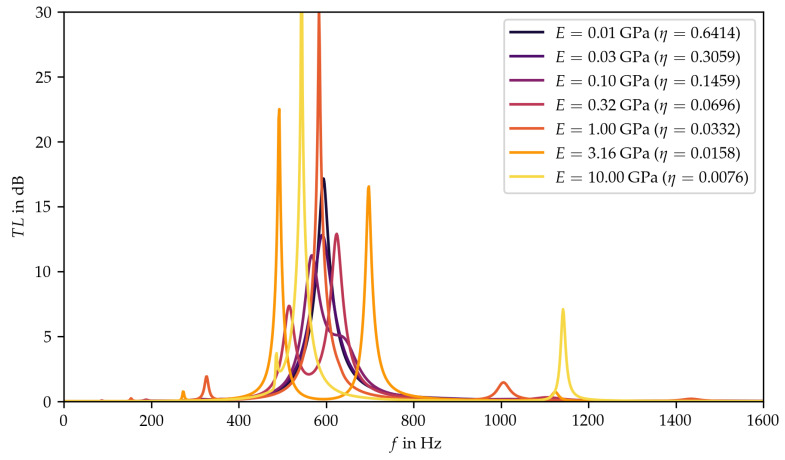
Results of the parameter study with functional dependence between Young’s modulus, E, and the corresponding loss factor, η.

In order to perform the parameter study, corresponding pairs of the material parameters with a logarithmic step size were selected, covering the whole value range of the targeted material group (Figure 4, orange diamonds). The key advantage of this approach is that the number of parameters to be included in the optimization process is reduced to one, since the corresponding value of the other parameter results from the determined functional relationship. The resulting chart displays the variation of the transmission loss for the target value range, where the effect of the parameter combination is not clearly apparent (see Figure 5).

**Figure 6:**
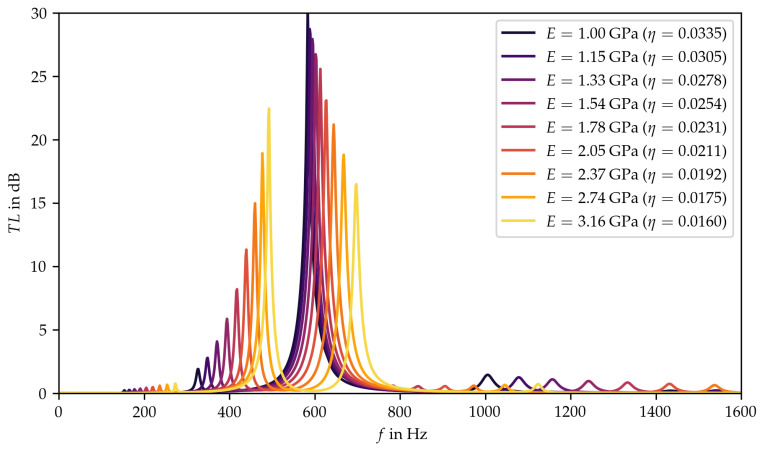
Results of the parameter study with reduced range and step size.

Therefore, the range and the step size between the parameter pairs was further reduced (Figure 4, red diamonds). In the corresponding chart, three effects become visible now (see Figure 6). On one side, the two main peaks were shifted to higher frequencies for higher values of the Young’s modulus. On the other side, the amplitude of the first main peak increased with increasing Young’s modulus, while the amplitude of the second peak decreased. Furthermore, the third effect showed that the frequency shift was not equidistant. Thus, the frequency shift steps of the first main peak became smaller as the Young’s modulus increased, while those of the second main peak became larger.

At this point, it would already be possible to proceed to the next step and select a material, since the influence of the Young’s modulus coupled with the loss factor on the transmission loss is readily apparent in the narrower parameter range. However, in the present case, there was a significant difference between the behavior of the transmission loss with a large step size of the Young’s modulus and a small step size. Thus, it seems to be worthwhile to investigate the whole target parameter range of the Young’s modulus linked with the loss factor with a finer resolution to gain a better understanding of the behavior of the plate silencer. Since this step requires a greater computational effort than decreasing the range with the step size (see Figure 6), it is not necessarily recommended within the framework of the material selection method.

**Figure 7:**
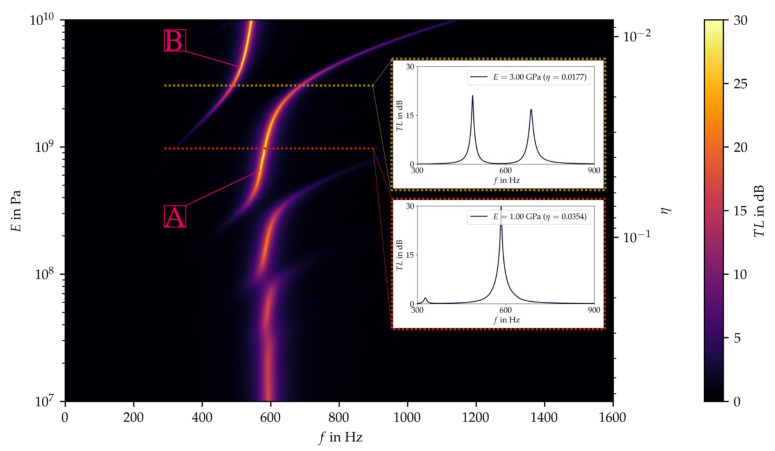
Results of the parameter study with a fine parameter resolution.

However, in order to support the formulation of an optimization goal and subsequently draw an elaborated conclusion on the material selection, a continuous spectral analysis showing the effect of the relevant parameters on the target variable over the entire parameter range of the material group could be beneficial. Since in the present case the computation time is reasonable using average computational resources, a more detailed calculation can be performed over the entire target value range. The results were presented in the form of a color map (see Figure 7), which is comparable to a topographic map. In this context, the yellow areas are the peaks of the transmission losses, as shown in the figures above. A cut through the color chart affords the transmission loss values over the frequency spectrum of a single value pair of Young’s modulus and loss factor. This corresponds to the type of plots already known (see Figure 6) and illustrates two sections inserted in the color map (Figure 7, red and yellow dashed line). It can be seen that, depending on the combination of Young’s modulus and loss factor, one or two peaks appear in transmission loss over the frequency spectrum. This observation is consistent with that in Figure 6, as the parameter range displayed there corresponds to the area between the yellow and the red dashed line in Figure 7.

In addition, s-shaped patterns became apparent in the color map (see Figure 7). These patterns are caused by the eigenmodes of the plate. Following the colored area starting from the lowest Young’s modulus at approximately 600 Hz to higher values of the Young’s modulus, the s-shaped form of the transmission loss over the frequency spectrum becomes more apparent. In between these s-patterns, discontinuities occurred, becoming more distinctive with increasing Young’s modulus. These discontinuities correspond to a shift of the eigenfrequency from a higher to a lower odd mode of the plate. This explains why no clear trend could be observed within the preliminary parameter study applying a wider step size (see Figure 5). In Addition, Figure 7 shows that the s-shaped patterns became more distinct and brighter with increasing Young’s modulus, and faded and became a little darker with increasing loss factor. This implies that for higher Young’s moduli, the peaks of the transmission loss become higher and narrower, whereas for higher loss factors, the peak broadens and the transmission loss decreases.

#### 3.2.5. Selection

Based on the observed trends, the specific Young’s modulus combined with the loss factor can now be selected to achieve the desired characteristics for the plate silencer. Thus, to realize the objective of low-frequency attenuation mentioned above, a high Young’s modulus is preferable, because higher transmission losses appear at lower frequencies for higher Young’s moduli. In contrast, lower Young’s moduli decrease the transmission losses and shift them to higher frequencies.

In order to select an optimum parameter range, it seems reasonable to move along curve A, because of the high transmission losses below 600 Hz. At a Young’s modulus of 1 MPa, a second curve B appeared at around 400 Hz. Thus, the mode shift between *E* = 1 and 3 MPa revealed a region with two peaks. Above a Young’s modulus of 3 MPa, curve A turned to higher frequencies outside the targeted range. In this range, curve B shows high transmission losses. However, curve A simultaneously shows strongly decreasing transmission losses in the frequency range up to 600 Hz. Due to this and the fact that in the present case two transmission loss peaks in a low-frequency range are considered superior to one, the optimal parameter range of the Young’s modulus was determined between approximately 1 and 3 MPa (Figure 7, between red and yellow dashed line).

At this point, a difference between the introduced method and the established procedure applying performance indices becomes apparent, where the optimization target needs to be defined at the beginning. In contrast, the present method provides a visual representation that affords a coherent perspective on the results for the whole range of parameters given by the functional dependence. This makes it possible to identify the effect of Young’s modulus and the corresponding loss factor on the transmission loss within the frequency spectrum and to adjust the performance target, if necessary, up to the end of the material selection process. As in this case, where two peaks at a lower frequency might be superior to one with a higher transmission loss, depending on the application.

**Figure 8:**
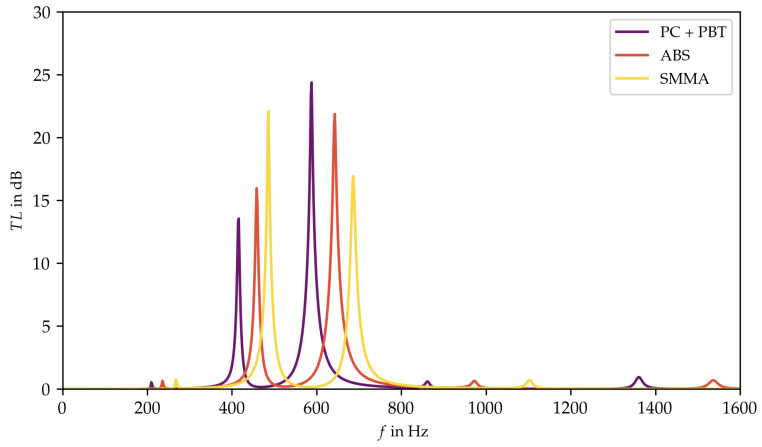
Transmission loss for specific plate materials [35].

Consequently, the range of 1 to 3 MPa provides the optimal parameter pairs of Young’s modulus and loss factor which are used to select real existing materials. Therefore, the materials that match the parameter pairs closely are identified in the material chart (see Figure 4). Since the real existing materials correspond only to a limited extent to the functional dependence, the transmission loss curves of the three selected materials: styrene methyl methacrylate (SMMA), acrylonitrile butadiene styrene (ABS), and polycarbonate + polybutylene terephthalate (PC + PBT), must be separately determined (see Figure 8) [35]. All three curves are qualitatively similar and show two distinct peaks. Thereby, PC + PBT achieved the attenuation at the lowest frequencies, which led to the selection of this material.

## 4. Conclusions

A new method of model-based material selection has been developed and successfully transferred to the application of the plate resonator. The method allows the visualization and coherent analysis of the influences on the target parameter over a certain value range by determining a functional dependency between relevant material parameters. By adjusting the step sizes and the display of the results, a method was established that can be applied to the process of material selection of similarly complex systems. In the presented example, the functional relationship represents the target parameter range well and allows a reduction of the parameter space. However, the accuracy of the functional relationship depends on the parameter range considered, which leads to a reduction of the material parameter range in case the accuracy of the functional relationship is low. Therefore, the method indeed greatly relies on the functional dependency between relevant material parameters of the targeted material class. Consequently, if no dependence can be determined, a coherent analysis of the wide parameter range cannot be performed. Considering this, the presented method has the potential to be applied where the precise formulation of an optimization target might be difficult. In this context, it supports the decision-making process by visualizing the effects of certain parameters on a target variable over a wide value range.

## Figures and Tables

**Table 1:** Values of geometry and material parameters for modeling a 2D plate silencer.

Parameter	Symbol	Unit	Value/Value Range	Design Point
Geometry parameter				
Cavity length	$l_{C}$	mm	60	60
Cavity height	$h_{C}$	mm	30	30
Cavity width	$w_{C}$	mm	∞	∞
Duct height	$h_{D}$	mm	60	60
Duct width	$w_{D}$	mm	∞	∞
Plate thickness	$h_{P}$	mm	0.3	0.3
Material parameter				
Young’s modulus	E	N/m^2^	E∈ {10^7^; 10^8^; 10^9^; 10^10^}	10^9^
Poisson ratio	ν	-	0.4	0.4
Loss modulus	η	-	η∈ {0.0015; 0.015; 0.15}	0.015
Density	ρ	kg/m^3^	ρ∈ {850; 950; 1050; 1150}	1050

## Data Availability

The data presented in the current study are available upon request from the corresponding author.
